# Postmortem diagnosis of diabetes mellitus and its complications

**DOI:** 10.3325/cmj.2015.56.181

**Published:** 2015-06

**Authors:** Cristian Palmiere

**Affiliations:** CURML, Centre Universitaire Romand De Medecine Legale, Lausanne University Hospital, Lausanne, Switzerland

## Abstract

Diabetes mellitus has become a major cause of death worldwide and diabetic ketoacidosis is the most common cause of death in children and adolescents with type 1 diabetes. Acute complications of diabetes mellitus as causes of death may be difficult to diagnose due to missing characteristic macroscopic and microscopic findings. Biochemical analyses, including vitreous glucose, blood (or alternative specimen) beta-hydroxybutyrate, and blood glycated hemoglobin determination, may complement postmortem investigations and provide useful information for determining the cause of death even in corpses with advanced decompositional changes. In this article, we performed a review of the literature pertaining to the diagnostic performance of classical and novel biochemical parameters that may be used in the forensic casework to identify disorders in glucose metabolism. We also present a review focusing on the usefulness of traditional and alternative specimens that can be sampled and subsequently analyzed to diagnose acute complications of diabetes mellitus as causes of death.

Diabetes mellitus has become a major cause of death worldwide in people younger than 60 years. Over the past three decades, the number of people with diabetes mellitus has more than doubled globally, making it one of the most important public health challenges to all nations (1). Worldwide, 382 million adults (8.3%) are living with diabetes, and the estimate is projected to rise to more than 592 million by 2035. At least US $147 billion was spent on diabetes health care in Europe in 2013, whereas North America and the Caribbean spent $263 billion (2). Currently, in Australia, approximately 4.0% of people aged 15 years and over has been diagnosed with diabetes. Some estimates suggest that this figure will rise to as much as 2 million by 2025 as a result of increasing obesity and aging as well as changes in the ethnic composition of the Australian population (2,3).

Type 1 diabetes is characterized by cellular-mediated autoimmune destruction of pancreatic beta-cells resulting in insulin deficiency and, thus, hyperglycemia (4,5). In the United States, Canada, and Europe, type 1 diabetes accounts for 5 to 10% of all cases of diabetes. A second and more prevalent category, type 2 diabetes, is characterized by a combination of insulin resistance and inadequate compensatory insulin secretory response (5,6).

Prevalence and morbidity data in cases of already diagnosed diabetes underestimate the actual burden of the disease since it is usually not diagnosed until it has become clinically apparent and complications occur. A number of local and national surveys have reported both diagnosed and undiagnosed diabetes rates based on population health surveys, though the relative prevalence of diagnosed and undiagnosed cases varies widely. The North-West Adelaide Health Survey, for example, found a ratio of 5–6:1 for diagnosed vs undiagnosed diabetes, consistent with the latest Australian Bureau of Statistics National Health Survey data showing a ratio of 5:1, whereas a previous Australian Diabetes, Obesity and Lifestyle Study estimated one undiagnosed case for every diagnosed case in Australia (3,7). Analogously, a high proportion of the estimated 26 million Americans with diabetes remains undiagnosed and unaware of their disease, and more than 90% of the estimated 79 million adults with pre-diabetes remains undetected (8,9).

Diabetic ketoacidosis (DKA) is a life-threatening condition that can occur when there is a complete lack of insulin, as in type 1 diabetes, or inadequate insulin levels associated with stress or severe illness in either type 1 or type 2 diabetes (10). It has been estimated that nearly a third of all deaths from DKA occurs in individuals with no known history of diabetes (6,11).

DKA is the most common cause of death in children and adolescents with type 1 diabetes and accounts for half of all deaths in diabetic patients younger than 24 years of age (12). Depending on the reports, DKA at the clinical diagnosis of type 1 diabetes in the pediatric population may range from 15% to more than 77% of cases (13).

In the realm of forensic pathology, acute complications of diabetes mellitus as causes of death may be difficult to diagnose due to missing characteristic macroscopic and microscopic findings. Nevertheless, when biochemical investigations complement autopsy and histology, fatal DKA can be easily diagnosed despite unknown disease history and even in corpses with advanced decompositional changes (6,14,15).

The aim of this article is to perform a review of the literature pertaining to the diagnostic performance of classical and novel biomarkers that may be used in forensic pathology routine to identify disorders in glucose metabolism. Moreover, we wish to present a review of the literature focusing on the usefulness of traditional and alternative specimens that can be sampled at autopsy and subsequently analyzed to diagnose acute complications of diabetes mellitus as causes of death.

## Vitreous glucose

In clinical practice, the most important biochemical markers to identify disorders in glucose metabolism are blood glucose concentration and glycated hemoglobin levels. In the realm of forensic pathology, postmortem blood glucose concentration is unreliable and of no diagnostic value in estimating antemortem blood glucose levels due to substantial fluctuations in glucose concentrations after death. After the cessation of cardiac and respiratory functions, surviving cells continue to metabolize blood glucose for some time and glycolysis continues spontaneously, causing a rapid decrease in blood glucose levels. Furthermore, death may be preceded by agonal processes and/or cardiopulmonary resuscitation, often associated with catecholamine release or administration. This results in subsequent mobilization of liver glycogen and increases in blood glucose concentrations as a counterbalancing phenomenon. Another possible pitfall in estimating antemortem blood glucose values using postmortem blood glucose concentrations is the variation of glucose levels depending on the sampling site. The highest blood glucose concentrations have been found in hepatic vein blood, followed by inferior vena cava, superior vena cava, and cardiac right ventricle blood, likely following glycogen breakdown in the liver. Considering the difficulties in interpreting glucose levels in postmortem blood, determination of glucose concentrations and those of its metabolites in biological fluids other than blood, such as vitreous humor and cerebrospinal fluid, was proposed in order to detect antemortem hyperglycemia (6,11,16-19).

Assuming that anaerobic glycolysis continues spontaneously after death and that two molecules of lactic acid are the final product of the postmortem glycolysis of one molecule of glucose, Traub (20) speculated that blood glucose concentrations at the time of death could be estimated by combining the values of glucose and lactate in cerebrospinal fluid

Blood glucose concentration at the time of death:

Glucose cerebrospinal fluid concentration + (Lactate cerebrospinal fluid concentration/2)

Succeeding Traub, other research teams have reasserted the applicability and reliability of this formula in vitreous humor or cerebrospinal fluid in order to predict glucose values shortly preceding death (21-26).

Karlovsek (27,28) compared glycated hemoglobin, glucose, lactate, and combined glucose and lactate concentrations in vitreous and cerebrospinal fluid in a series of medico-legal cases including diabetics and control individuals. Based on her results, Karlovsek proposed that vitreous glucose concentrations over 13 mmol/L (234 mg/dL) or combined glucose and lactate levels in vitreous humor or cerebrospinal fluid over 23.7 mmol/L (427 mg/dL) and 23.4 (422 mg/dL), respectively, could indicate antemortem hyperglycemia with a fatal outcome, thus suggesting that increased vitreous glucose levels alone were diagnostic of antemortem blood hyperglycemia. The additional measurements of glycated hemoglobin, acetone, acetoacetate, and beta-hydroxybutyrate were also recommended in order to confirm or exclude DKA as the cause of death.

Zilg et al (29) measured glucose and lactate in vitreous humor sampled as soon as possible after corpse arrival at the morgue in a large series of medico-legal cases. They observed that after an initial decrease in vitreous glucose levels during the very early postmortem period, glucose concentrations did not undergo substantial changes for an appreciable amount of time after death. Conversely, vitreous lactate levels constantly increased as postmortem interval lengthened, as did, consequently, the combined value of vitreous glucose and lactate. On the other hand, they (29) postulated that the presence of lactate in the vitreous could be due not only to postmortem glucose metabolism, but to other sources responsible for its formation and subsequent increased concentrations in the vitreous after death. Based on these findings, the authors postulated that vitreous glucose alone could be used to estimate blood glucose levels at the time of death. In addition, they proposed that vitreous glucose values over 10 mmol/L (180 mg/dL), theoretically corresponding to antemortem blood glucose concentrations of approximately 26 mmol/L (468 mg/dL), might indicate death due to acute complications of diabetes (DKA or hyperosmolar hyperglycemic states, based on ketone levels) or, in the least, that hyperglycemia might contribute to death.

Similar results were obtained by Palmiere et al (30) in a series of medico-legal cases including diabetics and control individuals. These authors concluded that vitreous glucose alone was more reliable than the sum value of vitreous (or cerebrospinal fluid) glucose and lactate in order to estimate antemortem blood glucose levels. They also reasserted the importance of simultaneous determination of blood glycated hemoglobin, acetone, and beta-hydroxybutyrate, as well as urine glucose, to better characterize the metabolic profile of individual cases and confirm (or rule out) DKA as cause of death.

In a review article published in 2011, Hess et al (17) concluded that, due to the considerable influence of environmental temperatures in glycolysis, the Traub formula was still up-to-date and should be considered when estimating antemortem glucose levels. However, these authors did emphasize that lactate values could be increased for reasons other than antemortem glucose metabolism disturbances, such as malignant tumors, alcohol-induced lactic acidosis, respiratory insufficiency, and inflammation. Nevertheless, in a paper published in 2013, Hess et al (6) concluded that post-mortem vitreous lactate determination did not seem to provide further information and could therefore be omitted.

In their recent study, Keltanen et al (31) took a balanced, mid-way position between the two opposing statements regarding the Traub formula reliability. They concluded that combined glucose and lactate values may be elevated in cases of antemortem hyperglycemia, though not exclusively in cases of antemortem hyperglycemia, and that the Traub formula should not be used for diagnosing hyperglycemia, though it does prove useful in interpreting vitreous glucose and lactate levels in cases with relatively long postmortem intervals and low vitreous glucose values, possibly due to prolonged postmortem glycolysis. Furthermore, they asserted that higher combined glucose and lactate values in the vitreous, though not diagnostic per se, should prompt pathologists to look for complementing postmortem biochemical investigations. Examples of these are glycated hemoglobin and beta-hydroxybutyrate determination, the latter being the most reliable indicator of ketoacidosis and its severity.

## Glycated hemoglobin and other glycated proteins

The glycation process results from a spontaneous reaction between the aldehyde group of a monosaccharide (usually glucose) and the free amine groups of peptides, such as hemoglobin, albumin, and other serum proteins. Glycated hemoglobin consists of several fractions that differ not only in the location of the glycated amine group but also in the type of attached monosaccharide. The biggest fraction of glycohemoglobin is, however, glycated hemoglobin HbA1c, produced by a two-step, irreversible, non-enzymatic, post-translational, spontaneous reaction between D-glucose and the N-terminal amine group of the hemoglobin β chain. The rate of this reaction is determined by the concentration of glucose in the blood (32).

HbA1c is the most widely used measurement in evaluating long-term glycemic control as well as the risk for complication development in patients with type 1 or 2 diabetes. Nevertheless, HbA1c determination is known to be effected by hematologic conditions that change erythrocyte survival or diseases characterized by variant hemoglobins, possibly responsible for erroneous glycemic control evaluation. Hyperglycemia is also known to reduce erythrocyte survival, thereby leading to an underestimation of HbA1c levels in diabetic patients with poor glycemic control (33-35).

In addition to hemoglobin, most serum proteins undergo non-enzymatic glycation in both euglycemic and diabetic patients in relation to the blood glucose levels they are exposed to in their life-spans. Measurements of glycated albumin and glycated total proteins, the latter commonly referred to as fructosamine, may be used as the determination of glycated hemoglobin. These measurements have therefore been proposed as useful tools for glycemic control evaluation in diabetics. The level of glycated albumin or glycated total proteins in plasma or serum reflects shorter-term glycemic control in diabetics because they have a shorter half-life than glycated hemoglobin. Serum glycated albumin has been reported as a useful, reliable indicator of glycemic control in diabetic patients since the serum albumin turnover is much shorter (17-day half-life) than that of HbA1c. Circulating albumin is strongly glycated at four sites of lysine residues, with the glycation reaction occurring ten times more rapidly than that of hemoglobin. Analogously, serum fructosamine, produced by the spontaneous, non-enzymatic glycation of serum proteins, has a shorter half-life than HbA1c and reflects very recent (1–3 weeks) glycemic control. This may potentially lessen the confounding effects of shortened red cell survival or high red cell turnover seen with HbA1c levels. Fructosamine concentration largely reflects glycated albumin concentration, even though a limited amount of fructosamine is made up of other serum proteins. Glycation of serum proteins with very short half-lives should reflect recent metabolic fluctuations in glycemic control even more than glycated hemoglobin, glycated albumin, and glycated total proteins (36-40).

In forensic pathology routine, HbA1c is a useful marker to characterize the metabolic state of the deceased in the weeks prior to death. HbA1c is measured in whole blood. Unlike glucose, it is fairly stable after death and corresponds well to antemortem values. Nonetheless, some discrepancies may arise when blood samples are markedly hemolyzed or putrefied (6,11,16,17,19,32,41-49).

Wineker et al (47) found no differences in HbA1c values if blood samples were stored in tubes with EDTA or sodium fluoride. Hindle et al (42) and Goullé et al (48) observed that HbA1c determination was possible in whole blood specimens stored at 4°C for about 40 days in samples collected with EDTA, for about 3 months in samples collected with sodium fluoride, and for 6 months in samples collected in dry or heparinized tubes.

Uemura et al (49) investigated a series of laboratory parameters, including glycated hemoglobin and fructosamine, in whole blood (for glycated hemoglobin) and postmortem serum (for fructosamine) obtained from three different sampling sites (left cardiac blood, right cardiac blood, and femoral vein blood) in a series of consecutive forensic autopsy cases. Though HbA1c values measured in femoral blood were lower than those measured in cardiac blood, both HbA1c and fructosamine could be reliably determined in specimens (whole blood or postmortem serum) obtained from any sampling site. Of all tested laboratory parameters, glycated hemoglobin showed the smallest deviation from living subjects, negligible postmortem changes, and no differences due to the cause of death. On the contrary, fructosamine showed a large deviation from living subjects. The authors postulated that different postmortem chemical behaviors of HbA1c and fructosamine could be related to the nature of the tested parameter. Indeed, hemoglobin glycation in the living is a cumulative process, whose rate depends on the level of prevailing glucose concentration under the lifespan of erythrocytes. Conversely, serum fructosamine concentrations reflect its normal destruction or elimination by functioning organs.

Glycated albumin and fructosamine in postmortem samples obtained from diabetics and control cases were investigated by several authors (50-53). John et al (50) and Akane et al (51,52) experienced significant difficulties in measuring fructosamine after death due to highly hemolyzed or extremely hemoconcentrated samples, which rendered the material unsuitable for analysis. Conversely, Valenzuela (53) obtained encouraging results from fructosamine assays, confirming its usefulness for the postmortem diagnosis of diabetes mellitus.

Ritz et al (40) studied *in vitro* α1-antitrypsin and haptoglobin glycation as well as glycated α1-antitrypsin and haptoglobin resistance to autolysis. They found that both proteins underwent *in vitro* glycation considerably more rapidly than either albumin or hemoglobin and that glycation proved to be highly resistant to autolysis. Glycated α1-antitrypsin and haptoglobin levels were further determined in blood obtained from living diabetics and non-diabetic control individuals as well as in blood obtained from diabetic cadavers. The results of these analyses revealed that glycated α1-antitrypsin and haptoglobin levels closely reflected blood glucose concentrations at the time of blood sampling (in the living) or shortly before death, thus indicating that the postmortem determination of both parameters might be a useful laboratory tool for the postmortem diagnosis of diabetes.

Lastly, only four studies evaluated glucose and fructosamine concentrations in vitreous humor samples obtained after death from diabetics and control cases. Osuna et al (54,55) and Vivero et al (56) noticed higher levels of both parameters in diabetics compared to control individuals. These results suggested that combined increased glucose and fructosamine levels in the vitreous might support the hypothesis of antemortem hyperglycemia and diabetes mellitus (6,54-57). Hess et al (6) observed significant differences in blood fructosamine concentrations between diabetics and non-diabetics. Conversely, no significant differences were found in cerebrospinal fluid and vitreous fructosamine levels.

## Acetone, acetoacetate, and beta-hydroxybutyrate

Ketone bodies, sometimes incorrectly called ketones, include three molecules: acetoacetate, beta-hydroxybutyrate, and acetone. Ketones are organic compounds that contain a chemical group consisting of a carbon atom double-bonded to an oxygen atom and also bonded to two other carbon atoms. Examples of ketones are pyruvate and fructose. Acetone and acetoacetate are both ketones and ketone bodies. Chemically speaking, beta-hydroxybutyrate is not a ketone, though it is classified as a ketone body because it exists in equilibrium with acetoacetate. Both acetoacetate and beta-hydroxybutyrate are acid anions, meaning that increased levels of these compounds result in a drop of blood pH and subsequent (keto)acidosis. Ketone bodies are a by-product of fat metabolism and are primarily synthesized in the liver as an alternative energy source. In diabetics, enhanced ketone body production is the consequence of the inability to use glucose due to insulin insufficiency or insulin resistance. In other physiological and pathological conditions, such as prolonged fasting, enhanced ketone body synthesis is the result of glucose unavailability. Ketone bodies are mainly produced within the mitochondria of hepatocytes, in particular within perivenous hepatocytes. Acetoacetate and beta-hydroxybutyrate are energy-rich compounds that transport energy from the liver to other tissues. They can be interconverted by the enzyme beta-hydroxybutyrate dehydrogenase. Acetone is generated through the decarboxylation of acetoacetate (either spontaneously or through the enzyme acetoacetate decarboxylase) and is generally considered of little metabolic significance. Increased levels of isopropyl alcohol, a ketone body-related compound, have been observed in numerous clinical conditions characterized by increased ketone body levels in blood and elevated NADH/NAD+ ratios. In these situations, isopropyl alcohol excess is thought to be the result of acetone metabolism and its conversion by the enzyme alcohol dehydrogenase (58-60).

Diabetes mellitus is the most common pathologic cause of increased ketone body production and subsequent ketoacidosis. The hallmark of DKA is relative insulin deficiency (type I diabetes) or a major increase in insulin resistance (type II diabetes), both resulting in hyperglycemia, whereas alcoholic ketoacidosis and starvation ketoacidosis are usually associated with reduced nutritional intake thereby accompanied by hypoglycemia (61-66).

Postmortem investigations of ketone bodies in blood and other biological fluids have been carried out by several researchers (18,19,61,62). Beta-hydroxybutyrate seems to be a better postmortem indicator of ketoacidosis than acetone (57,62).

Kanetake et al (67) measured postmortem serum ketone body levels in a series of autopsy cases including diabetics and alcoholics. These authors proposed that beta-hydroxybutyrate concentrations over 1000 µmol/L (corresponding to 10.4 mg/dL) might indicate ketoacidosis as the cause of death in situations characterized by enhanced ketone body production.

Iten and Meier (68) investigated blood concentrations of beta-hydroxybutyrate in cases of diabetics and alcoholic ketoacidosis. They proposed that blood beta-hydroxybutyrate concentrations up to 500 µmol/L (corresponding to 5.2 mg/dL) might be regarded as normal, from 500 to 2500 µmol/L (corresponding to 26 mg/dL) as increased, and over 2500 µmol/L as pathological.

Elliott et al (69) measured beta-hydroxybutyrate concentrations in blood, urine, and the vitreous in a large series of fatalities including diabetics, alcoholics, and diabetic alcoholics. In most cases, they observed comparable vitreous and blood beta-hydroxybutyrate values. Though urine beta-hydroxybutyrate levels were not perfectly equivalent to blood levels, they proposed the same interpretative range for blood, vitreous, and urine beta-hydroxybutyrate values in order to diagnose ketoacidosis after death (normal <50 mg/L, corresponding to 480 µmol/L; increased 51-249 mg/L, corresponding to 490-2390 µmol/L; pathologically significant: >250 mg/L, corresponding to 2400 µmol/L).

The findings of these studies were corroborated by our own investigations, which also confirmed that fatal DKA can be diagnosed using vitreous humor as an alternative to postmortem blood with a beta-hydroxybutyrate cut-off value of 2500 µmol/L. Besides the vitreous, our analyses indicated that pericardial fluid can also be reliably analyzed for beta-hydroxybutyrate determination should blood prove unavailable during autopsy (61).

The usefulness of vitreous beta-hydroxybutyrate determination for the postmortem diagnosis of diabetes mellitus has also been emphasized by Osuna et al (55) and Heniger (70). The latter author proposed a series of consecutive vitreous beta-hydroxybutyrate levels varying from normal (<400 µmol/L, corresponding to approximately 40 mg/L) to significantly elevated (2000-6000 µmol/L, 200-625 mg/L), and definitely indicating life-threatening conditions (>6000 µmol/L).

Biochemical investigations focusing on beta-hydroxybutyrate concentrations in blood, vitreous, urine, and cerebrospinal fluid in alcoholics and diabetics were carried out by Kadiš et al (71) and Felby et al (72). Kadiš et al (71) found that beta-hydroxybutyrate levels in cerebrospinal fluid were significantly lower than those in blood, vitreous, and urine. The authors postulated that such results were the consequence of low ketone body permeability in the blood-brain barrier and concluded that cerebrospinal fluid was not appropriate for postmortem beta-hydroxybutyrate determination. They also proposed a cut-off value of 3000 µmol/L (approximately 300 mg/L) in blood, urine, and vitreous to diagnose ketoacidosis. Felby et al (72) investigated beta-hydroxybutyrate concentrations in blood, vitreous, spinal fluid, and urine in a series of medico-legal autopsy cases and observed that urine had the lowest correlation with blood beta-hydroxybutyrate levels. Moreover, spinal fluid values were generally lower than blood and vitreous levels. Though analogous results had already been described by Kadiš et al (71), diverging conclusions were drawn by Felby et al (72) concerning the usefulness of spinal fluid in diagnosing ketoacidosis. Indeed, since spinal fluid is renewed more rapidly than the vitreous, Felby et al (72) concluded that spinal fluid was more reliable than the vitreous in reflecting the actual biochemical situation at the time of death.

Similar results were obtained by our group when we compared blood, vitreous, urine, and pericardial and cerebrospinal fluid beta-hydroxybutyrate levels in diabetics and alcoholics. Cerebrospinal fluid concentrations were mostly lower than blood levels, thereby indicating that cerebrospinal fluid beta-hydroxybutyrate levels over 2000 µmol/L (200 mg/L), possibly corresponding to 2500 µmol/L (260 mg/L) in blood, might be used to diagnose ketoacidosis after death. Urine beta-hydroxybutyrate values were usually higher than blood levels and significantly higher than blood levels on occasion. We therefore recommend cautiousness when interpreting increased beta-hydroxybutyrate urine levels using postmortem blood cut-off values.

Though less precise than beta-hydroxybutyrate in diagnosing ketoacidosis in the postmortem setting, acetone levels in diabetics and alcoholics have been investigated by some research teams in blood and alternative biological fluids (23,73-75). Pounder et al (73) measured levels of total ketone bodies in postmortem samples including the vitreous, pericardial fluid, and blood from different sampling sites. Vitreous ketone body levels showed good correlation with blood and pericardial fluid levels. Increased ketone body levels over 10000 µmol/L in blood (and over 5000 µmol/L in the vitreous) were indicative of severe ketoacidosis. Brinkmann et al (74) investigated blood acetone levels in a series of forensic cases and proposed a blood acetone cut-off value of 9 mg/dL (corresponding to 1.5 mmol/L) as indicative of fatal ketoacidosis.

Interesting results were obtained by Palmiere et al (76,77), who measured beta-hydroxybutyrate levels in liver homogenates and synovial fluid. Beta-hydroxybutyrate concentrations in liver homogenates correlated well with blood values in both diabetic and non-diabetic patients and were not influenced by time after death, thus allowing diabetic ketoacidosis to be diagnosed. Analogously, blood and synovial fluid beta-hydroxybutyrate concentrations were correlated with increased blood levels reflected in increased synovial fluid concentrations (76,77).

As far as the influence that postmortem interval may have on postmortem beta-hydroxybutyrate levels, Iten and Meier (68) analyzed the relationship between blood beta-hydroxybutyrate concentrations and time after death. No statistical increases in postmortem blood beta-hydroxybutyrate levels were observed. These findings led to the conclusion that decompositional changes were not associated with beta-hydroxybutyrate production and that blood beta-hydroxybutyrate levels in decomposed bodies could be considered an appropriate biochemical parameter in the estimation of beta-hydroxybutyrate concentrations at the time of death. Similar results were obtained in two studies, where we investigated blood beta-hydroxybutyrate levels in a series of medico-legal autopsies that included bodies with decompositional changes (15,78). Indeed, Kadiš et al (71) had already postulated that beta-hydroxybutyrate did not increase after death but, at most, decreased due to spontaneous molecule degradation.

## Isopropyl alcohol

The concomitant presence of acetone and isopropyl alcohol in biological samples from living and deceased people was commonly accepted as the result of direct exposure to isopropyl alcohol. However, Robertson et al (79) observed isopropyl alcohol in the blood, milk, and rumen contents of cows suffering from acetonemia and speculated that acetone could be converted to isopropyl alcohol.

Lewis et al (80) subsequently postulated that alcohol dehydrogenase could reduce acetone to isopropyl alcohol in certain situations, including diabetes mellitus. They studied normal and diabetic rats and concluded that isopropyl alcohol was a metabolic product of acetone, thereby providing an alternative explanation to direct isopropyl alcohol exposure for the increased isopropyl alcohol levels seen in humans.

Analogously, Davis et al (81) tested the hypothesis that in situations characterized by increased ketone body levels, the excessive amounts of acetone produced by the organism could enter a shunt mechanism resulting in its reduction to isopropyl alcohol. This might be especially true in the presence of an elevated NADH/NAD+ ratio, a biochemical feature of ketotic states. The authors documented the production of isopropyl alcohol from acetone *in vitro* in the presence of the enzyme alcohol dehydrogenase and also reported increased acetone and isopropyl alcohol levels in the blood and tissues (liver, brain, and kidney) of 8 individuals who had not been exposed to isopropyl alcohol. The highest isopropyl alcohol concentrations were noted in the liver, with isopropyl alcohol values exceeding those of acetone in that organ alone.

Buszewicz and Mądro (82) investigated the *in vitro* reduction of acetone to isopropyl alcohol in human homogenates of the liver, brain, and lungs. They concluded that despite extremely significant individual differences, the acetone-isopropyl alcohol conversion was a near-equimolar process, allowing the estimation of initial acetone concentrations via the summation of the molar concentrations of the two substances.

Isopropyl alcohol is currently considered a marker of ketoacidosis and a product of acetone metabolism in clinical conditions presenting increased ketone body levels. The compound can be detected in several situations of forensic interest, beyond direct exposure to isopropyl alcohol itself, characterized by increased acetone levels and an elevated NADH/NAD+ ratio. These conditions include diabetic and alcoholic ketoacidosis as well as hypothermia fatalities and starvation (78).

The results of isopropyl alcohol determination in cases of sudden death in diabetics have been reported among others by Teresiński et al (75), with measured concentrations in femoral blood ranging from 1 to 15 µmol/L (0.06-0.90 mg/L), and Palmiere et al (78), with measured concentrations in femoral blood ranging from 330 to 1577 µmol/L (20-95 mg/L). In the latter study, isopropyl alcohol was also measured in urine and vitreous humor at concentrations ranging from 365 to 847 µmol/L (22-51 mg/L) and from 282 to 813 µmol/L (17-49 mg/L), respectively.

Significantly higher blood and vitreous isopropyl alcohol concentrations in diabetics were found by Molina (83), with measured blood values ranging from 0 to 50 mg/dL (median value 11.5 mg/dL) and vitreous values ranging from 0 to 50 mg/dL (median value 8 mg/dL), and Petersen et al (84), who identified an average concentration of isopropyl alcohol of 15.1 ± 13.0 mg/dL in a series of 175 DKA cases.

## C-reactive protein

Besides the immediate biochemical derangements, such as the pathologically elevated glucose and ketone body levels that characterize the onset of DKA and are ultimately responsible for death, an increasing number of clinical studies have recently focused on biochemical markers that can identify or reveal the presence and progression of acute complications of DKA (essentially pulmonary and cerebral edema). These markers included C-reactive protein, high sensitivity C-reactive protein, interleukin 6, interleukin 10, interleukin 2, interleukin 4, interleukin 8, interleukin 1α, interleukin 1β, and tumor necrosis factor alpha (TNF-α) (14).

In the realm of forensic pathology, C-reactive protein levels in diabetic and alcoholic ketoacidosis have been investigated by Lindroos-Jokinen et al (85). These authors observed that C-reactive protein levels could successfully be measured in postmortem material up to 18 days after death. Furthermore, they noted that ketoacidosis itself was associated with increased C-reactive protein levels without any other obvious, underlying causes. These might include infection or trauma, which typically lead to higher C-reactive protein concentrations.

Analogously, Palmiere et al (14) observed increased C-reactive protein, interleukin 6, and interleukin 10 levels in a series of fatal DKA cases, without any macroscopic or microscopic signs of bacterial infection. Moreover, in most of these cases, procalcitonin and lipopolysaccharide binding protein concentrations were at normal levels, thus corroborating the hypothesis of enhanced acute inflammatory responses in the course of DKA in the absence of obvious bacterial infection.

## Urine glucose

At euglycemic blood glucose concentrations, glucose is freely filtered at the glomerulus and completely reabsorbed at the level of the proximal convoluted tubule. With rising blood glucose, the reabsorption of filtered glucose in the proximal convoluted tubule increases until a maximum value is reached. Any further increase in blood glucose (and in the resultant glucose load presented to the proximal tubule) results in the excretion of glucose in urine. The appearance of glucose in urine is reflected in the concept of a renal threshold for glucose excretion. Theoretically, no glucose should be detectable in urine at sub-threshold blood glucose levels. Nevertheless, the excretion of glucose in urine in small amounts at euglycemic or sub-threshold blood glucose levels represents a phenomenon described in the literature as basal glycosuria. Basal or physiological glycosuria is independent of blood glucose concentration, urinary flow rates, renal threshold for glucose, and maximal rate of tubular glucose absorption. These features suggest that physiological glycosuria does not reflect active transport system capacity in the proximal tubules, but may very well be the result of distal tubular leakage (86).

Although glucose is detectable in the urine of patients with increased blood glucose concentrations, its value in urine gives no information about blood glucose levels below the renal threshold for glucose excretion, which vary considerably among individuals.

In the realm of forensic pathology, urine glucose in diabetics was studied by some research teams (6,17). Though high glucose values in urine were demonstrated only in cases of diabetic coma, glucose concentrations in urine were weakly correlated with glucose levels in vitreous and cerebrospinal fluid (6,17). These observations led to the conclusion that glucose value determination in urine is of low significance for the postmortem detection of diabetic coma. Increased urine glucose levels may be indicative of marked hyperglycemia. However, low urine glucose concentrations do not allow this hypothesis to be excluded. Hence, urinary glucose should be exclusively used to confirm consistent findings obtained from vitreous glucose and blood ketone body measurements (6,15,17).

Mitchell et al (87) recently evaluated the usefulness of a rapid screening test in urine (urine “dipstick” testing using test strips) for the detection of increased glucose and ketone body levels. They found that urinalysis (“dipstick” testing) for glucose provided high specificity for high vitreous glucose levels, irrespective of the urinary result threshold. Conversely, urinalysis for ketone bodies provided either excellent sensitivity with low specificity or poor sensitivity with good specificity, depending on the urinary result threshold. According to the authors, these findings were likely due to the fact that test strips indicate levels of acetoacetate, while the predominant ketone body of pathogenic ketotic states is beta-hydroxybutyrate.

## Urea nitrogen, creatinine, urate, sodium, and chloride

Few fatal cases of hyperosmolar hyperlgycemic states (HHS) have been reported in the forensic literature. HHS is typically associated with type 2 diabetes and is characterized by hyperglycemia, absent or small ketonuria, low ketonemia, hyperosmolarity, and profound dehydration. Both DKA and HHS result from a reduction in the amount or effective action of circulating serum insulin, producing intracellular starvation. The responding counterregulatory mechanisms result in a state of hyperglycemia and lipolysis, with subsequent hepatic fatty acid oxidation producing ketone bodies. In HHS, however, plasma insulin concentrations may still be adequate to prevent lipolysis and subsequent ketogenesis, resulting in marked hyperglycemia, osmotic diuresis, and dehydration. The patient may experience profound water and electrolyte loss before presentation. If not managed properly, the risks in this compromised hemodynamic state include severe electrolyte disturbance, arrhythmia, rhabdomyolysis, renal failure, cardiovascular collapse, cerebral edema, and death (46,62,88,89). According to Hockenhull et al (62), death as a result of HHS is characterized by high glucose concentrations, indicating hyperglycemia at the time of death, with no significant amount of ketone bodies detected.

Among the various biochemical markers investigated in the realm of forensic pathology, the results of numerous studies have demonstrated that urea nitrogen, creatinine, and uric acid are relatively stable in postmortem serum collected during autopsy. These may therefore be used for diagnostic purposes when dehydration and impaired renal function are investigated. Pericardial fluid urea nitrogen, creatinine, and urate have been demonstrated as relatively stable within 48 hours postmortem, with levels unrelated to postmortem intervals. Moreover, urea nitrogen, creatinine, and urate concentrations in pericardial fluid are independent of sampled volume amounts, indicating no significant interference of postmortem water redistribution. Based on the results of these studies, it was postulated that pericardial fluid urea nitrogen, creatinine, and urate may be regarded as suitable biochemical markers for the pathophysiological investigation of situations in which dehydration and impaired renal function might be involved and postmortem serum unavailable (90-93).

Vitreous sodium levels have been determined to be relatively stable during the early postmortem period and similar to levels found in the serum of living subjects. According to the literature, abnormalities in antemortem serum sodium concentrations are reflected in postmortem vitreous values (16). Like sodium, vitreous chloride concentrations show minimal falls in values during the early postmortem period and abnormalities in antemortem serum chloride are reflected in postmortem vitreous values (19).

## Conclusions

The identification of acute complications of diabetes mellitus as causes of death may be extremely challenging in forensic pathology routine due to the absence of specific signs at autopsy and histology. It has been repeatedly emphasized that biochemical analyses, especially vitreous glucose determination, should systematically complement postmortem investigations in all unexplained deaths. What has been shown in recent years is that biochemical analyses can be performed after death even in corpses with advanced decompositional changes, using alternative specimens. Results obtained from these analyses can still provide useful data for determining the cause of death.

Another point that deserves to be highlighted is that, apart from cases of fatal DKA in individuals with previously undiagnosed diabetes mellitus, numerous situations of forensic interest may concern unintentional mismanagement of medical treatment (either insulin or hypoglycemic agents) in diabetics due to impaired judgment and/or unconsciousness caused by concomitant drug (either recreational or therapeutic) intoxication or potentially incapacitating diseases. This means that postmortem biochemical investigations should be systematically performed in both diagnosed and undiagnosed individuals in order to identify fatal complications of diabetes mellitus not only in all unexplained deaths with negative autopsy and toxicology, but even when autopsy and toxicology results seem to provide apparently decisive findings.

To conclude, diabetes mellitus is expected to become one of the most serious health problems in the world in the near future. Clinical pathologists, forensic pathologists, and forensic toxicologists must be aware of the diagnostic potential and limits of the available analyses and techniques. Determination of vitreous (or cerebrospinal fluid) glucose, blood (or alternative specimen) beta-hydroxybutyrate, and blood glycated hemoglobin are the standard analyses that any forensic laboratory should be able to carry out in order to detect potentially fatal disorders in glucose metabolism. Indeed, these analyses may allow DKA and HHS to be easily identified in both diagnosed and undiagnosed diabetics and a conclusive cause of death to be established in situations that would have otherwise remained unexplained.
